# Metabolic Signatures Differentiate Rett Syndrome From Unaffected Siblings

**DOI:** 10.3389/fnint.2020.00007

**Published:** 2020-02-25

**Authors:** Jeffrey L. Neul, Steven A. Skinner, Fran Annese, Jane Lane, Peter Heydemann, Mary Jones, Walter E. Kaufmann, Daniel G. Glaze, Alan K. Percy

**Affiliations:** ^1^Vanderbilt University Medical Center, Nashville, TN, United States; ^2^Department of Neurosciences, University of California, San Diego, San Diego, CA, United States; ^3^Baylor College of Medicine, Houston, TX, United States; ^4^Greenwood Genetic Center, Greenwood, SC, United States; ^5^Department of Pediatrics, University of Alabama at Birmingham, Birmingham, AL, United States; ^6^Rush University Medical Center, Chicago, IL, United States; ^7^Benioff Children’s Hospital Oakland, University of California, San Francisco, San Francisco, CA, United States

**Keywords:** urea cycle, neurodevelopmental disorders, biomarker (development), MeCP2, metabolomics (OMICS), rett syndrome, Kreb’s cycle enzymes, amino acids

## Abstract

Rett syndrome (RTT, OMIM 312750), a severe neurodevelopmental disorder characterized by regression with loss of spoken language and hand skills, development of characteristic hand stereotypies, and gait dysfunction, is primarily caused by *de novo* mutations in the X-linked gene *Methyl-CpG-binding protein 2* (*MECP2*). Currently, treatment options are limited to symptomatic management, however, reversal of disease phenotype is possible in mouse models by restoration of normal *MECP2* gene expression. A significant challenge is the lack of biomarkers of disease state, disease severity, or treatment response. Using a non-targeted metabolomic approach we evaluated metabolite profiles in plasma from thirty-four people with RTT compared to thirty-seven unaffected age- and gender-matched siblings. We identified sixty-six significantly altered metabolites that cluster broadly into amino acid, nitrogen handling, and exogenous substance pathways. RTT disease metabolite and metabolic pathways abnormalities point to evidence of oxidative stress, mitochondrial dysfunction, and alterations in gut microflora. These observed changes provide insight into underlying pathological mechanisms and the foundation for biomarker discovery of disease severity biomarkers.

## Introduction

Rett syndrome (RTT, OMIM 312750) is a neurodevelopmental disorder that primarily affects girls and is usually caused by mutation in the X-linked gene *Methyl-CpG-binding Protein 2* (*MECP2*) (Amir et al., 1999; Neul et al., 2008). Affected individuals usually have a normal birth and apparently normal initial development, followed by developmental stagnation and then regression of acquired spoken language and hand skills with the development of characteristic repetitive hand stereotypies and gait problems (Neul et al., 2010). Individuals with RTT also have a variety of additional clinical features including seizures, movement abnormalities, growth failure, gastro-intestinal problems, and autonomic dysfunction (reviewed in Neul, 2011). Currently approaches to therapies are symptomatic, however work in mouse models provides hope that targeted therapies hold promise of significantly modifying or even reversing the disease (Guy et al., 2007). Recently, promising clinical trials in RTT have been completed (Glaze et al., 2017, 2019) or are being initiated, that could alter the treatment options in this disease.

There exists a need for biomarkers in RTT. First, evaluation of molecular or neurophysiological biomarkers might provide insight into the underlying pathophysiology of disease. Second, biomarkers of disease severity could be useful in clinical trials as early markers of treatment response. Finally, with the onset of potential disease modifying therapies, there is a need for early detection of affected individuals. Because most cases of RTT are caused by *de novo* mutations in *MECP2* (Amir et al., 1999), there is no established family risk profile. Additionally, most people with RTT are not diagnosed until after regression. Disease biomarkers could provide additional information on disease state allowing for earlier diagnosis and intervention.

Previous work evaluating metabolite abnormalities in a targeted fashion have found a variety of abnormal features in RTT. Evaluation of spinal fluid identified decreased biogenic amine metabolites (Samaco et al., 2009). A variety of reports have found molecular evidence of oxidative in red blood cells, blood, and patient-derived fibroblasts in people with RTT, as well as in mouse models of RTT (reviewed in Shulyakova et al., 2017; Muller, 2019). To date however, no large scale non-targeted metabolomics studies have been reported in RTT. Metabolomics, the measurement of small molecules such as endogenous metabolites, peptides, xenobiotics, dietary components, and agents of environmental exposure, is one of the newest and rapidly developing “-omics” fields but has already proven to be very useful in a variety of contexts including characterizing age and gender changes in the metabolome of adults (Lawton et al., 2008) and finding metabolomic changes in ALS (Lawton et al., 2012). Metabolomics describes the dynamic cellular “phenotype,” integrating transcription, protein function, and environmental factors to bridge to organismal phenotype.

To capitalize on the power of untargeted metabolomic analysis, we characterized a cohort of individuals with RTT and their unaffected gender- and age-matched siblings using a well-established commercial platform (Metabolon, NC, United States). A number of metabolites and metabolic pathways that differentiate affected from unaffected individuals were identified providing insight into underlying disease processes in RTT. The metabolite differences may also be useful as either disease state or severity biomarkers.

## Methods

### Human Subjects

Subjects were recruited from the RTT Natural History Study (RNHS), RTT5201; CT.gov: **NCT00299312**. The RNHS is part of the Rare Diseases Clinical Research Network (RDCRN), established through the Office of Rare Diseases Research, National Center for Advancing Translational Sciences at the National Institutes of Health. All participants in the RNHS were required either to meet clinical criteria for RTT (Neul et al., 2010) and/or to have a mutation in *MECP2*. An experienced RNHS neurologist or geneticist (DGG, SAS, WEK, JLN, and AKP) with extensive clinical experience in RTT utilized the established criteria for diagnosis of RTT or other related phenotypes. Clinical information was stored in a de-identified fashion in a centralized database. For this study, blood samples were acquired under a related institutional review board protocol at Baylor College of Medicine (BCM Protocol H-26509). Subjects enrolled in RNHS and unaffected family members were recruited and blood was drawn in standard clinical fashion. Samples were collected from non-fasted individuals throughout the day. Plasma was immediately separated and stored at −80°C until sent in a de-identified fashion to Metabolon (Morrisville, NC, United States)^1^. For this study, samples from 34 individuals with RTT and 37 unaffected gender and age (±2 years) matched siblings were analyzed (Supplementary Table S1).

### Metabolomic Analysis

De-identified samples were shipped on dry ice to Metabolon (Morrisville, NC, United States^1^) for analysis. Samples were analyzed using a Liquid Chromatography-Tandem Mass Spectrometry (LC-MS/MS) platform and a Gas Chromatography-Mass Spectroscopy (GC-MS) platform. The LC-MS portion of the platform was based on a Waters ACQUITY ultra-performance liquid chromatography (UPLC) and a Thermo-Finnigan LTQ mass spectrometer operated at nominal mass resolution, which consisted of an electrospray ionization (ESI) source and linear ion-trap (LIT) mass analyzer. The MS analysis alternated between MS and data-dependent MS/MS scans using dynamic exclusion and the scan range was from 80 to 1000 m/z. The GC-MS portion was analyzed on a Thermo-Finnigan Trace DSQ fast-scanning single-quadrupole mass spectrometer using electron impact ionization (EI) and operated at unit mass resolving power. The scan range was from 50–750 m/z. Raw data was extracted, peak-identified and QC processed using Metabolon’s hardware and software. Compounds were identified by comparison to library entries of purified standards or recurrent unknown entities. Metabolon maintains a library based on authenticated standards that contains the retention time/index (RI), mass to charge ratio (m/z), and chromatographic data (including MS/MS spectral data) on all molecules present in the library. Metabolite peaks were quantified using area-under-the-curve. Missing values were imputed using the minimum observed value for each compound.

### Statistical Analysis

All analysis was performed using MetaboAnalyst 4.0^2^ (Xia and Wishart, 2016), a comprehensive web-based application for metabolic data analysis and interpretation. A companion R based MetaboAnalyst package has also been created (Chong and Xia, 2018), and R script for all analyses done in this manuscript is provided. The data file provided from Metabolon was uploaded to MetaboAnalyst and no filtering was applied. Values were log transformed and mean centering data scaling was applied (full normalized data set provided in Supplementary Table S2). Fold change (RTT/unaffected siblings) and log2 Fold change calculated for graphical presentations. A *t*-test was performed for each metabolite (comparing RTT to unaffected sibling, unpaired) and uncorrected *p*-values and to control for multiple testing a False Discovery Rate (FDR) corrected *p*-values calculated. The full table of all *t*-test and fold change results is presented in Supplementary Table S3.

Hierarchical clustering analysis was performed in MetaboAnalyst using hclust function in package stat, using the 25 most significantly different metabolites (lowest *p*-values), with Euclidean distance and Ward’s linkage. The results were then plotted as a heat map showing the clusters. Random Forest (RF) analysis, a supervised learning algorithm for high dimensional data analysis was performing using randomForest package in MetaboAnalyst with 500 trees. During tree construction, 1/3 of instances were left out of the bootstrap sample for out-of-bag classification error and Mean Decrease Accuracy was calculated for each metabolite. The RF features are presented in Supplementary Table S4. The R-script for the *t*-test, fold analysis, hierarchical clustering, and RF analysis is presented in Supplementary Material S1.

The Biomarker module of MetaboAnalyst was used to generated Receiver Operator Characteristic (ROC)-curve based assessments of biomarkers that best discriminated between affected and unaffected individuals. The same processing of the data was performed as in the *t*-test analysis, and ratios of the top 20 metabolites were also calculated. Classical ROC analysis was performed on each metabolite or combined metabolite ratio pair and the area-under-the-curve (AUC), *t*-test, sensitivity and specificity calculated. The full table from the ROC analysis is presented in Supplementary Table S5 and the R-history in Supplementary Material S2.

Pathway analysis was performed using the MetPA pathway enrichment module in MetaboAnalyst matching to Human Metabolite Database (HDMB) IDs and human KEGG pathway library. Over-representation analysis was performed using hypergeometric test with relative betweenness centrality node importance measure for topological analysis. Metabolites with uncorrected *p* < 0.1 were included in the pathway analyses. The raw *p*-value plus Holm-Bonferroni and False Discovery Rate corrected *p*-values were calculated, with an Impact Value calculated from pathway topology analysis and presented in Supplementary Table S6, with the name mapping for KEGG analysis in Supplementary Table S7 and the R-history in Supplementary Material S3. Metabolic Set Enrichment Analysis (MSEA) was also used to evaluate for pathway over-representation using the MetaboAnalyst module. The Small Molecule Pathway Database (SMPDB) library was used for the analysis, and hypergeometric testing for the over-representation analysis. The complete results and name map are presented in Supplementary Tables S8, S9, with the R-history in Supplementary Material S4.

All graphs were generated in MetaboAnalyst or in Microsoft Excel.

## Results

Plasma samples were collected from 34 individuals with RTT and 37 unaffected gender and age (±2 years) matched siblings (Supplementary Table S1). Samples were collected from two siblings for three affected subjects, the remaining had one sibling each. Thirty-two subjects had classic RTT and two had variant (or atypical) RTT. Subjects had a variety of *MECP2* mutations, with 55.9% common hot-spot point mutations (R106W, R133C, T158M, R168X, R255X, R270X, R294X, and R306C). Age ranged between 3.4 and 25.0 years, with an average of 11.3 years old. Overall clinical severity, as assessed by the RTT Clinical Severity Score (CSS) (Neul et al., 2008), averaged 23.5 with a range of 11–41. This range and average severity is representative of severity ranges typically found in the RNHS (Cuddapah et al., 2014). Body Mass Index (BMI) and BMI percentage (BMI%) also ranged from very low to the high end of expected BMI for typically developing individuals (Supplementary Table S1), a distribution also often seen in RTT populations (Tarquinio et al., 2012).

**FIGURE 1:**
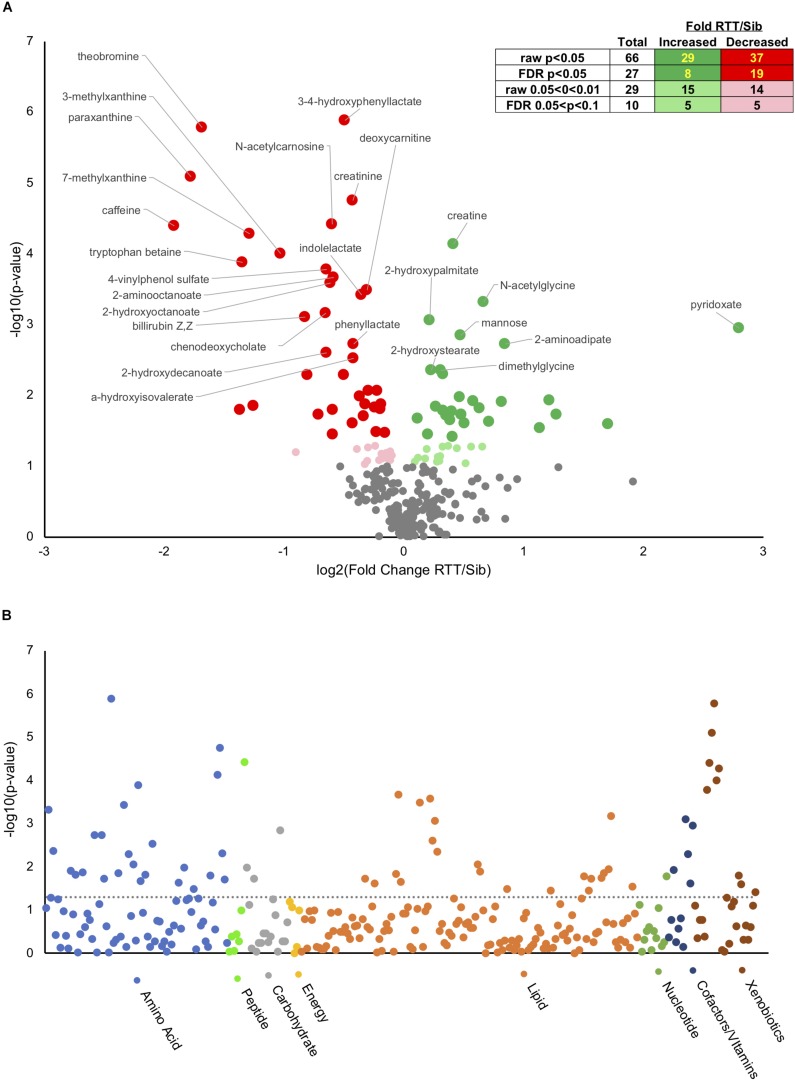
Metabolic changes in RTT compared with unaffected siblings. **(A)** Volcano plot showing negative log10(*p*-values) on *y*-axis and log2(Fold change RTT/sibling) on *x*-axis. Inset table shows number of metabolites at different raw or FDR corrected *p*-value and the distribution between increased in RTT compared to siblings or decreased. Dark green, increased *p* < 0.05, light green increased 0.05 < *p* < 0.1. Dark red, decreased *p* < 0.05, pink decreased 0.05 < *p* < 0.1. **(B)** Manhattan plot displaying all metabolites characterized arranged by chemical groups on the *x*-axis, with the *y*-axis showing the negative log10(*p*-value) for each metabolite. The gray line indicates uncorrected *p* = 0.05. Color indicates chemical groupings as indicated along the *x*-axis.

Analysis of metabolites using the Metabolon platformed identified 295 named compounds of known identity (Supplementary Table S2). Of these, 66 were different at an uncorrected *p*-value, and 27 different with a False Discovery Rate (FDR) corrected *p* < 0.05 (Figure 1A, insert). Of the 66 significantly different metabolites, 29 of which were increased in affected compared to unaffected and 37 decreased (Figure 1A inset and Supplementary Table S10). An additional 29 compounds showed a trend (*p* < 0.1 raw *p*-value) between affected an unaffected individuals, 15 of which were increased and 14 decreased in people with RTT compared with unaffected siblings. Figure 1A displays a volcano plot of all the *p*-values and fold changes, with the FDR significant metabolites labeled. Figure 1B shows a Manhattan plot grouping all compounds observed by chemical category. A number of changes were observed in xenobiotics (such as caffeine and related metabolites) that likely reflect differences in oral consumption between affected and unaffected individuals as most (11/14) were decreased in affected individuals. In contrast, nearly half of changes observed in amino acids and lipids were increased in affected individuals, suggesting that these differences might reflect underlying pathological processes in RTT.

**FIGURE 2:**
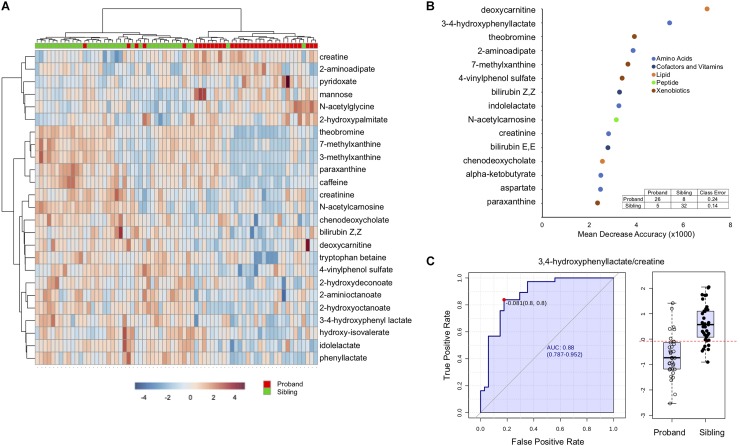
Discriminating features between affected and unaffected individuals. **(A)** Hierarchical clustering of the top 25 significantly different metabolites. **(B)** Top 15 metabolites distinguishing affected from unaffected as determined by Random Forest Analysis. The *x*-axis is the mean decrease accuracy for the metabolite (value × 1000). Metabolite groupings are indicated by colors identified in the legend. The inset shows the out of box based confusion matrix for the random forest classification. **(C)** The left side of the figure demonstrates the ROC curve for 3,4-hydroxyphenyl acetate/creatine to distinguish affected from unaffected, with the sensitivity on the *y*-axis and the specificity on the *x*-axis. The right panel is a box-plot of the metabolite ratio for the two groups, with the boxed values indicating quartiles, and the red dotted line indicating the optimal cutoff for classification.

Hierarchical clustering, a method to create similar groups, was performed using the twenty-five (Figure 2A). Not all probands and siblings clustered together although the majority did, and clear patterns of groups of metabolites that were either up or down in probands compared to unaffected siblings. To identify metabolites most important in classifying disease state, Random Forest (RF) Analysis was used and the top 15 metabolites are presented in Figure 2B. The performance of the RF Classification was good, as shown by the confusion matrix in Figure 2B, and the Out-of-bag error (OOB) of 0.183. Xenobiotics such as caffeine metabolites are again some of the most important classifying metabolites, however 11/15 are not xenobiotics and the top metabolites are deoxycarnitine (a metabolite of GABA and a precursor of carnitine) and 3,4-hydroxyphenyl lactate (a tyrosine metabolite). Furthermore, a number of metabolites are part of amino acid metabolism. To identify whether any single metabolite, or ratio of metabolites, might function as a biomarker to predict the disease state of an individual, we performed receiver operator characteristic (ROC) analysis. The feature (metabolite or ratio) with the highest area under the curve in the ROC curve analysis was 3,4-hydroxyphenyl lactate/creatine (Figure 2C), with an AUC of 0.88, and a jointly maximized sensitivity and specificity of 0.8/0.8. An optimal cutoff (Figure 2C, right panel) of this ratio to determine disease state shows reasonable, but not perfect, discrimination of affected an unaffected individuals.

**FIGURE 3:**
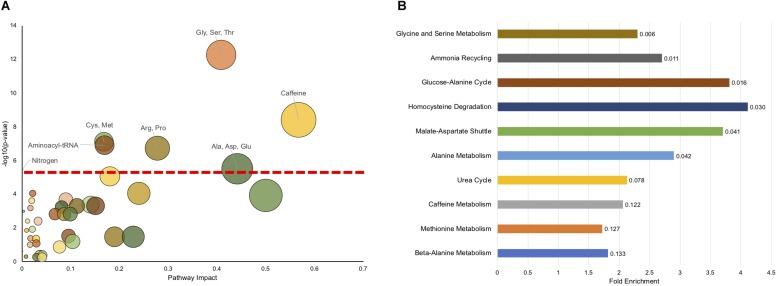
Pathway enrichment shows significant enrichment in metabolic pathways related to amino acid metabolism. **(A)** The impact on a pathway (Pathway Impact) for a given KEGG pathway is shown on the *x*-axis, and the −log(p) of the uncorrected *p*-value is presented on the *y*-axis. The dashed horizontal red line represents the *p* < 0.05 cutoff after FDR correction. The pathways that are significant (FDR < 0.05) are labeled. **(B)** MSEA overrepresentation analysis displaying the top 10 enriched pathways. The length of the bar is the fold enrichment of the pathway with the scale presented at the bottom. The numbers indicated the raw *p*-value for enrichment.

To see if there are any metabolic pathways that are enriched, we assessed KEGG pathway over-representation with metabolites that were different at a *p* < 0.1 level. Of the 95 metabolites with *p* < 0.1 (Supplementary Table S10), 83 were able to be linked to a unique Human Metabolome Database (HMDB) number for the analysis. Twenty pathways showed enrichment with uncorrected *p* < 0.05 (Supplementary Table S7), with seven having *p* < 0.05 after FDR correction. Figure 3A shows all the KEGG pathways graphed by uncorrected -log10(*p*-value) and pathway impact. As has been observed above, caffeine metabolism is enriched reflecting dietary differences between affected and unaffected, however, many metabolic pathways related to amino acid metabolism are also enriched, as is synthetic pathways important for tRNA synthesis and nitrogen metabolism.

We also used another approach to look for pathway enrichment using Metabolic Set Enrichment Analysis (MSEA), which is an adaptation of Gene Set Enrichment Analysis for metabolites. Six pathways showed enrichment with uncorrected *p* < 0.05, although none showed enrichment using FDR correction (Figure 3B). Again, amino acid metabolism (Glycine and Serine, Alanine) were enriched, as was homocysteine metabolism. Interestingly, there was also enrichment in ammonia recycling and urea cycle. Although these pathways were not identified using this exact classification in KEGG analysis, nitrogen metabolism was found to be significantly enriched and encompasses some of the same metabolites and pathways as found in urea cycle and ammonia recycling. Surprisingly, caffeine metabolism only trended (*p* = 0.122) toward significance using MSEA analysis.

It is interesting that aside from caffeine metabolism, the major enriched pathways are related to amino acid metabolism, as recent reports have found alterations in amino acid metabolism in other neurodevelopmental disorders, notably autism (Smith et al., 2019). Of the 20 key protein component amino acids, four were significantly different (*p* < 0.05), with aspartate and glutamate increased in RTT and arginine and histidine decreased (Supplementary Figure S1). Four additional amino acids trended toward significance (*p* < 0.1), with cysteine, glycine, and serine increased in RTT and phenylalanine decreased.

**FIGURE 4:**
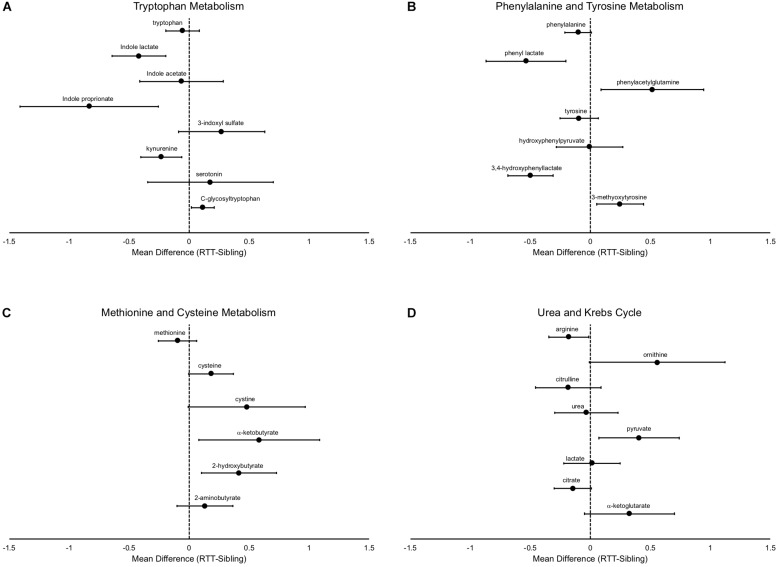
Specific metabolic differences between RTT and unaffected siblings. **(A)** shows alterations in tryptophan metabolism, **(B)** shows alterations in phenylalanine and tyrosine metabolism, **(C)** shows alterations in methionine and cysteine metabolism, and **(D)** in components of the urea and Krebs cycle. In all panels, the differences of the mean metabolite values between RTT and unaffected siblings is plotted with error bars representing the 95% confidence intervals.

In addition to differences in the amino acids themselves, there are notable differences in the metabolic pathways, even in pathways in which the primary amino acid itself is not changed. For example, tryptophan was not different between affected and unaffected siblings, however, a number of metabolites were changed (Figure 4A), notably decreased indole lactate, indole proprionate, and kynurenine. Similarly, although phenylalanine only showed a trend toward decrease in RTT and no differences were observed in tyrosine, a number of metabolites of these amino acids were altered (Figure 4B). Interestingly, a number of the metabolite abnormalities observed for tryptophan, phenylalanine, and tyrosine are metabolites that are primarily produced by gut microflora (De Angelis et al., 2015; Mussap et al., 2016). Methionine levels were similar between affected and unaffected individuals, but cysteine (and cystine) both trended toward increase (Figure 4C). Interestingly, two important metabolites produced during the production of cysteine, α-ketobutyrate and 2-hydroxybutyrate (also known as α-hydroxybutyrate) were increased in RTT subjects compared to siblings.

Arginine is decreased in subjects with RTT, however ornithine, which is converted by arginase from arginine, is increased (Figure 4D). In contrast, citrulline, which is the next product in the urea cycle, is numerically decreased in RTT subjects and urea levels are similar between affected an unaffected individuals, suggesting a complex alteration of the urea cycle. Citrate levels trended lower and α-ketoglutarate levels trended higher in RTT subjects, pointing toward alterations in the Krebs cycle. Pyruvate, a key supplier of acetyl CoA to the Krebs cycle, was increased, but lactate unchanged.

## Discussion

Systematic, broad, and non-targeted analysis of metabolites revealed distinct patterns that differentiate individuals affected with RTT from unaffected siblings. Although a number of the observed differences in metabolic pathways reflect likely dietary differences, such as caffeine and plant product metabolites, this work revealed a variety of other metabolites and metabolic pathways likely not related to dietary differences between affected and unaffected individuals. These differences provide both opportunities for biomarkers of RTT disease state, as well as insight into alterations in metabolism underlying pathogenic processes in RTT. Although previous work using targeted analysis has identified various metabolic abnormalities in people with RTT (Shulyakova et al., 2017; Muller, 2019), a clear strength of this work is the use of non-targeted analysis that allows for discovery of previously unrecognized changes to metabolic pathways.

Previous work has identified evidence for increased oxidative stress in RTT and suggested that this may reflect mitochondrial abnormalities. Specifically, in RTT subjects there have been found evidence of lipid peroxidation (Sierra et al., 2001), esterified isoprostanes (De Felice et al., 2009, 2011, 2012), plasma non-protein-bound iron (De Felice et al., 2009), and 4-hydroxynoneanal protein adducts (Ciccoli et al., 2012) and reduced glutathione in skin fibroblast cell lines derived from RTT subjects. Similar metabolite alterations have been seen in brains of RTT mouse models (De Felice et al., 2014; Szczesna et al., 2014). Although these specific metabolites were not measured in this work, we found evidence of alteration of key metabolic pathways that occur in the mitochondria, the Krebs cycle and the urea cycle. Additionally, there have been studies identifying abnormalities in the carnitine cycle in RTT, which occurs within mitochondria. In fact, treatment with levocarnitine can improve symptoms in people with RTT (Ellaway et al., 1999) and animal models (Schaevitz et al., 2012), and recent work has identified alterations in the expression of cardiac enzymes involved in the carnitine cycle in RTT mice (Mucerino et al., 2017). We observed changes in deoxycarnitine, a precursor of carnitine synthesis, and further exploration of these pathways is warranted.

Additionally, there is evidence of alterations in the methionine/cysteine metabolic pathway, with decreased levels of methionine and increased cysteine. In situations of increased oxidative stress, homocysteine is diverted from production of methionine to produce cystathione and ultimately cysteine to replenish glutathione levels. This results in increased production of α-ketobutyrate and 2-hydroxybutyrate (Gall et al., 2010), both of which were found to be markedly elevated in the RTT subjects assessed here suggesting an increased demand for glutathione in people with RTT due to increased oxidative stress and lipid oxidation, as implicated previously. In contrast, there was decreased levels of cysteine-glutathione disulfide, a molecule that is produced upon oxidative stress of glutathione. Future analysis would benefit from more detailed analysis of additional components of this pathway including homocysteine.

Glucose was found to be elevated in RTT subjects. Work in mouse models has identified insulin resistance and evidence of metabolic syndrome (Pitcher et al., 2013), and this plasma elevation of glucose could represent a similar unrecognized issue in people with RTT. The observed elevations in RTT subjects of 2-hydroxybutyrate and aminoadipate are supportive of this notion as elevations of these metabolites are biomarkers for pre-diabetes and diabetes (Li et al., 2009; Wang et al., 2013). Interestingly, aminoadipate is also a marker of oxidative stress (Yuan et al., 2011; Zeitoun-Ghandour et al., 2011). Another metabolite abnormality indicative of abnormal glucose levels is 1,5-anhydroglucitol, a sugar primarily derived from dietary sources whose reabsorption in the kidneys is competed by elevated levels of glucose (Parrinello and Selvin, 2014). The decreased levels observed in RTT subjects could be due to hyperglycemia, however, this finding could reflect the known dietary differences in these individuals. Nonetheless, the finding of increased markers (2-hydroxybutyrate and aminoadipate) in RTT subjects and evidence of insulin resistance in animal models warrants additional clinical monitoring of diabetes in RTT.

Some of the metabolite changes observed in the RTT subjects are similar to those observed in normal aging. C-glycosyl tryptophan increases with age (Menni et al., 2013), and was elevated in RTT subjects compared to sibling controls. Both 1,5-anhydroglucitol and the anti-oxidant N-acetyl carnosine levels decrease with age (Chaleckis et al., 2016) and were decreased in RTT subjects. It has been proposed that these age-related changes may reflect alterations in the ability to handle oxidative stress or alterations of the urea cycle in elderly compared to younger individuals (Chaleckis et al., 2016). N-acetyl carnosine has been formulated into eye drops to help ameliorate lipid peroxidation in the lens and treat cataracts (Babizhayev et al., 2014), although a recent Cochrane review failed to find convincing evidence of efficacy (Dubois and Bastawrous, 2017). These results are suggestive that people with RTT may have evidence of accelerated aging.

Two metabolites of tyrosine metabolism were found to be changed in RTT subjects. Increased plasma levels of 3-methoxytyrosine, as observed in RTT subjects, has been found in people with aromatic amino acid decarboxylase deficiency (AADC). People with AADC have developmental delay, hypotonia, and movement abnormalities associated with decreased serotonin and dopamine (Hyland et al., 1992), and similar clinical and biochemical findings have been observed in RTT individuals and RTT mouse models (Samaco et al., 2009). 3,4-hydroxyphenyl lactate is also a tyrosine metabolite that is elevated in metabolic diseases such as phenylketonuria (Spaapen et al., 1987). We observed decreased levels of 3,4-hydroxyphenyl lactate in RTT. The D-form is produced by gut microflora, and this decrease may reflect changes in gut microflora constitution in RTT compared with unaffected siblings (Spaapen et al., 1987). 3,4-hydroxyphenyl lactate can also function as a natural anti-oxidant (Beloborodova et al., 2012), and the decreased levels of this metabolite in RTT may contribute to the overall increased oxidative stress observed.

There are other changes observed that may reflect alterations in gut microflora. Notably, two tryptophan metabolites, indolepropionate and indolelactate and produced by gut microflora (Clostridum sporogenes specifically) (Wikoff et al., 2009; Dodd et al., 2017) and are decreased in RTT subjects. Indolepropionate also acts as an antioxidant (Reiter et al., 1998). Tryptophan is metabolized via two major pathways, either through the indole pathway or through kynurenine. Surprisingly, kynurenine was also found to be markedly decreased in RTT subjects. Alterations in the kynurenine pathway have been found in a variety of neurological disorders such as Alzheimer’s Disease, Parkinson Disease, Multiple Sclerosis, and Amyotrophic Lateral Sclerosis (Lovelace et al., 2017), and the kynurenine system has been implicated in mitochondrial function and oxidative stress (Sas et al., 2018). Metabolites of kynurenine have opposing effects on neuronal excitation, with kynurenic acid acting as a neuroprotective agent by antagonizing NMDA receptors, and quinolinic acid acting as an NMDA agonist. Interestingly, aminoadipate, which is increased in RTT subjects, acts to inhibit the production of kynurenic acid (Wu et al., 1995). A significant question is whether these metabolic changes observed may contribute to the observed alteration in excitation/inhibition balance in animal models of RTT (Banerjee et al., 2019). More detailed and targeted analysis of the components of the kynurenine pathway in RTT are needed to gain insight into the consequences of reduced plasma levels of kynurenine.

## Limitations

Although this work benefits from the non-targeted metabolomics approach utilized, there are clear limitations. The primary limitation is that samples were collected from non-fasted subjects and time of collection was not controlled. It is well known that diet, especially recent food intake, and time of day can have marked effects on metabolic profiles. Future work should attempt to either control for these factors (diet, collection time) or capture this information to include in analysis. The other main limitation is that the current analysis only identified 295 named compounds and many key metabolic intermediates were not assessed. Future work could benefit from using newer platforms that can assess a larger number of metabolites, and the use of more detailed analysis targeting specific pathways of interest identified in this study. Finally, a limitation is that the metabolites were only identified using a single platform and not validated using an orthogonal method or on independent samples. Future work will entail validation in independent samples.

## Conclusion and Future Work

This work represents that only non-targeted metabolomics analysis done to date in RTT and revealed specific metabolic abnormalities and pathways associated with disease state. These findings provide the foundation for future analysis and confirmation of metabolite and metabolic pathway abnormalities in RTT that could serve as biomarkers of disease state. Future work will focus on more detailed analysis of these pathways and confirmatory characterization. A critical need is to identify molecular biomarkers of disease severity in RTT, and future work will focus on evaluation of larger numbers of affected individuals to identify such biomarkers. Additionally, similar evaluation of metabolic profiles from mouse models of RTT would strengthen the discovery of useful biomarkers. Although the majority of people with RTT have mutations in *MECP2*, mutations in other genes have been found to cause RTT (Sajan et al., 2017), and an interesting question is whether these individuals share similar metabolic changes observed here. Finally, it would be interesting to observe metabolic changes that occur during the course of treatment, especially treatments that provide factors critical to metabolic functioning (Ellaway et al., 1999; Glaze et al., 2009; Schaevitz et al., 2012).

## Data Availability Statement

All datasets generated for this study are included in the article Supplementary Material.

## Ethics Statement

The studies involving human participants were reviewed and approved by Baylor College of Medicine Institutional Review Board. Written informed consent to participate in this study was provided by the participants’ legal guardian/next of kin.

## Author Contributions

JN, AP, DG, WK, and SS contributed to the conception and design of the study. JN, SS, FA, JL, PH, MJ, WK, DG, and AP acquired and contributed to the organization of the materials used in study. JN performed all analysis. JN, AP, WK, and DG interpreted data. JN wrote first draft of the manuscript. All authors contributed to manuscript revision, read, and approved the submitted version.

## Conflict of Interest

The authors declare that the research was conducted in the absence of any commercial or financial relationships that could be construed as a potential conflict of interest.

## Footnotes

1 www.metabolon.com

2 www.metaboanalyst.ca

## Supplementary Material

FIGURE S1 Amino acid differences between RTT and unaffected siblings. The differences of the mean metabolite values between RTT and unaffected siblings is plotted with error bars representing the 95% confidence intervals.

TABLE S1 Subject characteristics.

TABLE S2 Complete normalized metabolite levels.

TABLE S3 List of *t*-test results and fold changes for all metabolites.

TABLE S4 Random Forest Features.

TABLE S5 Biomarker ROC results.

TABLE S6 KEGG Over-representation analysis results.

TABLE S7 KEGG analysis name matching.

TABLE S8 MSEA results.

TABLE S9 MSEA name matching.

TABLE S10 Significant metabolite features.

SUPPLEMENTARY MATERIAL S1 R-history for *t*-tests, HC, RF.

SUPPLEMENTARY MATERIAL S2 R-history for ROC.

SUPPLEMENTARY MATERIAL S3 R-history for KEGG ORA.

SUPPLEMENTARY MATERIAL S4 R-history for MSEA ORA.
