# Supplementary figures and images for: Metabolic Signatures Differentiate Rett Syndrome From Unaffected Siblings
Source: Front Integr Neurosci. 2020 Feb 25;14:7. doi: 10.3389/fnint.2020.00007 (PMC7052375; doi:10.3389/fnint.2020.00007)

## Slide 1
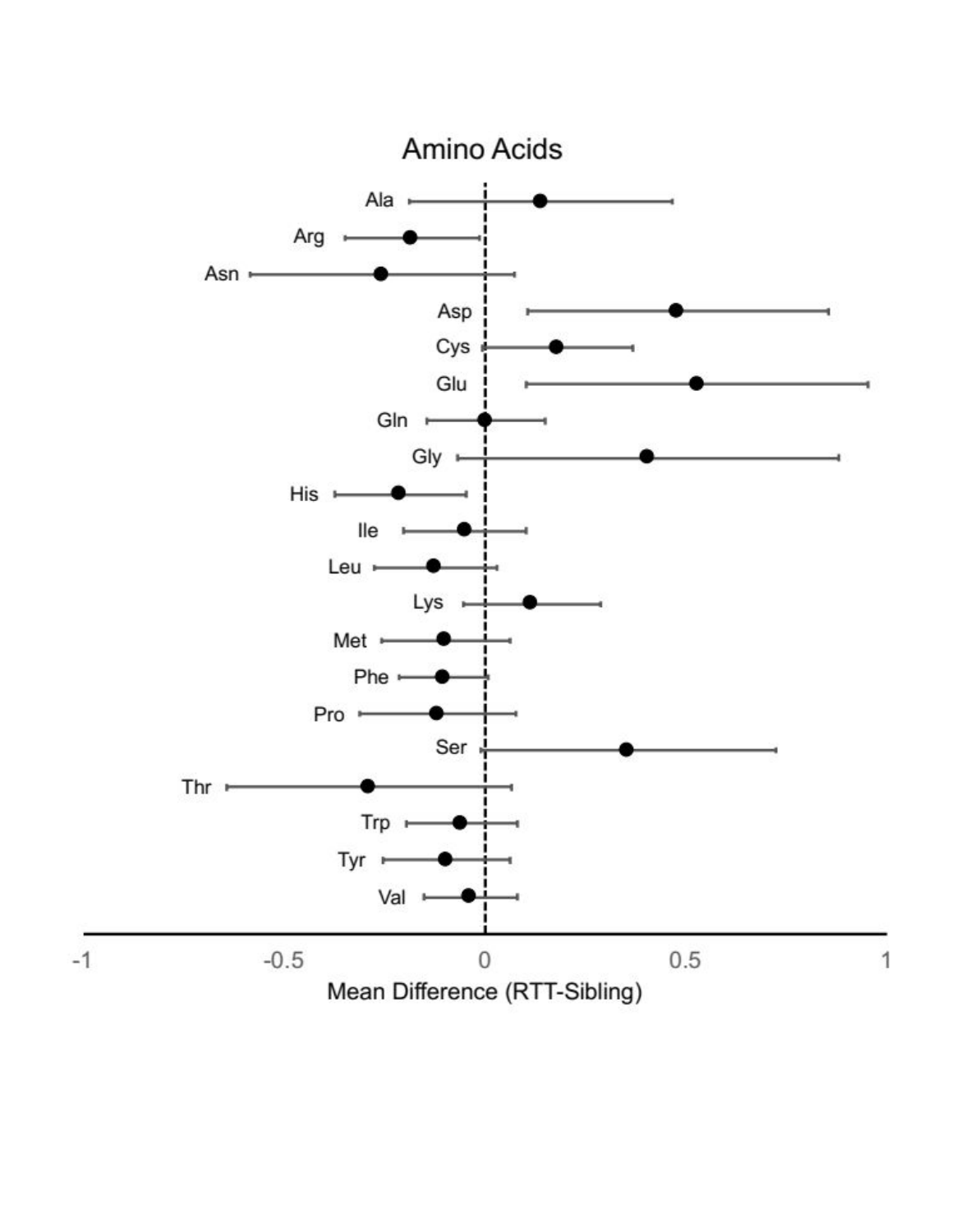

Supplement: FIGURE S1 — Amino acid differences between RTT and unaffected siblings. The differences of the mean metabolite values between RTT and unaffected siblings is plotted with error bars representing the 95% confidence intervals. [file Presentation_1.PPTX]
