# Supplementary material for: Metabolic Signatures Differentiate Rett Syndrome From Unaffected Siblings
Source: Front Integr Neurosci. 2020 Feb 25;14:7. doi: 10.3389/fnint.2020.00007 (PMC7052375; doi:10.3389/fnint.2020.00007)
Supplement: SUPPLEMENTARY MATERIAL S1 — R-history for t-tests, HC, RF. [file Data_Sheet_1.PDF]

```
mSet<-InitDataObjects("conc", "stat", FALSE)
mSet<-Read.TextData(mSet, "Replacing_with_your_file_path", "rowu",
"disc");
mSet<-SanityCheckData(mSet)
mSet<-ReplaceMin(mSet);
mSet<-FilterVariable(mSet, "none", "F", 25)
mSet<-PreparePrenormData(mSet)
mSet<-Normalization(mSet, "MedianNorm", "LogNorm", "MeanCenter",
ratio=FALSE, ratioNum=20)
mSet<-PlotNormSummary(mSet, "norm_0_", "png", 72, width=NA)
mSet<-PlotSampleNormSummary(mSet, "snorm_0_", "png", 72, width=NA)
mSet<-FC.Anal.unpaired(mSet, 2.0, 0)
mSet<-PlotFC(mSet, "fc_0_", "png", 72, width=NA)
mSet<-FC.Anal.unpaired(mSet, 1.0, 0)
mSet<-PlotFC(mSet, "fc_1_", "png", 72, width=NA)
mSet<-Ttests.Anal(mSet, F, 0.05, FALSE, TRUE)
mSet<-PlotTT(mSet, "tt_0_", "png", 72, width=NA)
mSet<-Ttests.Anal(mSet, F, 1.0, FALSE, TRUE)
mSet<-PlotTT(mSet, "tt_1_", "png", 72, width=NA)
mSet<-Volcano.Anal(mSet, FALSE, 2.0, 0, 0.75,F, 0.1, TRUE, "raw")
mSet<-PlotVolcano(mSet, "volcano_0_",1, "png", 72, width=NA)
mSet<-Volcano.Anal(mSet, FALSE, 1.0, 0, 0.75,F, 1.0, TRUE, "raw")
mSet<-PlotVolcano(mSet, "volcano_1_",1, "png", 72, width=NA)
mSet<-PlotVolcano(mSet, "volcano_1_",1, "png", 600, width=NA)
mSet<-PlotFC(mSet, "fc_1_", "png", 600, width=NA)
mSet<-SaveTransformedData(mSet)
mSet<-PlotHCTree(mSet, "tree_0_", "png", 72, width=NA, "euclidean",
"ward.D")
mSet<-PlotHCTree(mSet, "tree_0_", "png", 600, width=NA, "euclidean",
"ward.D")
mSet<-PlotHeatMap(mSet, "heatmap_0_", "png", 72, width=NA, "norm",
"row", "euclidean", "ward.D","bwm", "overview", T, T, NA, T, F)
mSet<-PlotHeatMap(mSet, "heatmap_0_", "png", 600, width=NA, "norm",
"row", "euclidean", "ward.D","bwm", "overview", T, T, NA, T, F)
mSet<-PlotSubHeatMap(mSet, "heatmap_1_", "png", 72, width=NA, "norm",
"row", "euclidean", "ward.D","bwm", "tanova", 25, "overview", T, T, T,
F)
mSet<-PlotSubHeatMap(mSet, "heatmap_1_", "png", 600, width=NA, "norm",
"row", "euclidean", "ward.D","bwm", "tanova", 25, "overview", T, T, T,
F)
mSet<-PlotSubHeatMap(mSet, "heatmap_1_", "png", 600, width=NA, "norm",
"row", "euclidean", "ward.D","bwm", "tanova", 25, "overview", T, T, T,
F)
mSet<-RF.Anal(mSet, 500,7,1)
mSet<-PlotRF.Classify(mSet, "rf_cls_0_", "png", 72, width=NA)
mSet<-PlotRF.VIP(mSet, "rf_imp_0_", "png", 72, width=NA)
mSet<-PlotRF.Outlier(mSet, "rf_outlier_0_", "png", 72, width=NA)
mSet<-PlotRF.VIP(mSet, "rf_imp_0_", "png", 600, width=NA)
```
