# Supplementary material for: Metabolic Signatures Differentiate Rett Syndrome From Unaffected Siblings
Source: Front Integr Neurosci. 2020 Feb 25;14:7. doi: 10.3389/fnint.2020.00007 (PMC7052375; doi:10.3389/fnint.2020.00007)
Supplement: SUPPLEMENTARY MATERIAL S2 — R-history for ROC. [file Data_Sheet_2.PDF]

```
mSet<-InitDataObjects("conc", "roc", FALSE)
mSet<-Read.TextData(mSet, "Replacing_with_your_file_path", "rowu",
"disc");
mSet<-SanityCheckData(mSet)
mSet<-ReplaceMin(mSet);
mSet<-FilterVariable(mSet, "none", "F", 25)
mSet<-PreparePrenormData(mSet)
mSet<-Normalization(mSet, "NULL", "LogNorm", "MeanCenter",
ratio=FALSE, ratioNum=20)
mSet<-PlotNormSummary(mSet, "norm_0_", "png", 72, width=NA)
mSet<-PlotSampleNormSummary(mSet, "snorm_0_", "png", 72, width=NA)
mSet<-SetAnalysisMode(mSet, "univ")
mSet<-PrepareROCData(mSet)
mSet<-CalculateFeatureRanking(mSet)
mSet<-SetAnalysisMode(mSet, "univ")
mSet<-PrepareROCData(mSet)
mSet<-SetAnalysisMode(mSet, "univ")
mSet<-PrepareROCData(mSet)
mSet<-FilterVariable(mSet, "none", "F", 25)
mSet<-PreparePrenormData(mSet)
mSet<-Normalization(mSet, "NULL", "LogNorm", "MeanCenter", ratio=TRUE,
ratioNum=20)
mSet<-PlotNormSummary(mSet, "norm_1_", "png", 72, width=NA)
mSet<-PlotSampleNormSummary(mSet, "snorm_1_", "png", 72, width=NA)
mSet<-SetAnalysisMode(mSet, "univ")
mSet<-PrepareROCData(mSet)
mSet<-CalculateFeatureRanking(mSet)
mSet<-Perform.UnivROC(mSet, "3-4-hydroxyphenyllactate/creatine", "3-4-
hydroxyphenyllactate_creatine_0_", "png", 72, F, T, "closest.topleft",
F, "sp", 0.2)
mSet<-PlotBoxPlot(mSet, "3-4-hydroxyphenyllactate_creatineboxplot",
"3-4-hydroxyphenyllactate_creatineboxplot_0_", "png", 72, T, FALSE)
mSet<-Perform.UnivROC(mSet, "3-4-hydroxyphenyllactate/creatine", "3-4-
hydroxyphenyllactate_creatine_0_", "png", 600, F, T,
"closest.topleft", F, "sp", 0.2)
mSet<-PlotBoxPlot(mSet, "3-4-hydroxyphenyllactate_creatineboxplot",
"3-4-hydroxyphenyllactate_creatineboxplot_0_", "png", 600, T, FALSE)
mSet<-SaveTransformedData(mSet)
```
