# Supplementary material for: Metabolic Signatures Differentiate Rett Syndrome From Unaffected Siblings
Source: Front Integr Neurosci. 2020 Feb 25;14:7. doi: 10.3389/fnint.2020.00007 (PMC7052375; doi:10.3389/fnint.2020.00007)
Supplement: SUPPLEMENTARY MATERIAL S3 — R-history for KEGG ORA. [file Data_Sheet_3.pdf]

```
mSet<-InitDataObjects("conc", "pathora", FALSE)
cmpd.vec<-
c("HMDB01847","HMDB01860","HMDB02825","HMDB94696","HMDB0061115","HMDB0
1991","HMDB00619","HMDB01886","HMDB00054","HMDB02302","HMDB02759","HMD
B00518","HMDB04072","HMDB94656","HMDB00711","HMDB12881","HMDB00365","H
MDB04827","HMDB00991","HMDB00755","HMDB01008","HMDB00562","HMDB00407",
"HMDB00779","HMDB02712","HMDB00671","HMDB00656","HMDB61700","HMDB01161
","HMDB10386","HMDB94649","HMDB0011341","HMDB00684","HMDB00695","HMDB0
0177","HMDB00517","HMDB37847","HMDB00131","HMDB31057","HMDB62549","HMD
B01434","HMDB00092","HMDB61384","HMDB02802","HMDB02925","HMDB0002013",
"HMDB61880","HMDB11753","HMDB00064","HMDB00008","HMDB00169","HMDB00243
","HMDB06344","HMDB05060","HMDB00210","HMDB00148","HMDB00532","HMDB000
05","HMDB00191","HMDB00510","HMDB00760","HMDB00036","HMDB01881","HMDB0
0017","HMDB29377","HMDB00258","HMDB00222","HMDB0008659","HMDB10379","H
MDB00625","HMDB61699","HMDB00725","HMDB00714","HMDB00094","HMDB00357",
"HMDB33433","HMDB01015","HMDB00159","HMDB00767","HMDB00122","HMDB15109
","HMDB00574","HMDB00705","HMDB00157","HMDB0061714","HMDB00208","HMDB0
1348","HMDB00063","HMDB00271","HMDB00187","HMDB00123","HMDB03374","HMD
B00192")
mSet<-Setup.MapData(mSet, cmpd.vec);
mSet<-CrossReferencing(mSet, "hmdb");
mSet<-CreateMappingResultTable(mSet)
mSet<-SetKEGG.PathLib(mSet, "hsa")
mSet<-SetMetabolomeFilter(mSet, F);
mSet<-CalculateOraScore(mSet, "rbc", "hyperg")
mSet<-PlotPathSummary(mSet, "path_view_0_", "png", 72, width=NA)
mSet<-PlotPathSummary(mSet, "path_view_0_", "png", 600, width=NA)
mSet<-PlotKEGGPath(mSet, "Glycine, serine and threonine metabolism",
528, 480, "png", NULL)
mSet<-RerenderMetPAGraph(mSet, "zoom1559581062824.png",528.0, 480.0,
100.0)
mSet<-PlotKEGGPath(mSet, "Glycine, serine and threonine
metabolism",NA, NA, "png", 600)
mSet<-PlotKEGGPath(mSet, "Caffeine metabolism",528, 480, "png", NULL)
mSet<-PlotKEGGPath(mSet, "Caffeine metabolism",NA, NA, "png", 600)
mSet<-PlotKEGGPath(mSet, "Alanine, aspartate and glutamate
metabolism",528, 480, "png", NULL)
mSet<-PlotKEGGPath(mSet, "Alanine, aspartate and glutamate
metabolism",NA, NA, "png", 600)
mSet<-PlotKEGGPath(mSet, "D-Arginine and D-ornithine metabolism",528,
480, "png", NULL)
mSet<-PlotKEGGPath(mSet, "D-Arginine and D-ornithine metabolism",NA,
NA, "png", 600)
mSet<-PlotKEGGPath(mSet, "Arginine and proline metabolism",528, 480,
"png", NULL)
mSet<-PlotKEGGPath(mSet, "Arginine and proline metabolism",NA, NA,
"png", 600)
mSet<-PlotKEGGPath(mSet, "Aminoacyl-tRNA biosynthesis",528, 480,
"png", NULL)
mSet<-PlotKEGGPath(mSet, "Aminoacyl-tRNA biosynthesis",NA, NA, "png",
```

```
600)
mSet<-PlotKEGGPath(mSet, "Cysteine and methionine metabolism",528,
480, "png", NULL)
mSet<-PlotKEGGPath(mSet, "Cysteine and methionine metabolism",NA, NA,
"png", 600)
mSet<-PlotKEGGPath(mSet, "Pantothenate and CoA biosynthesis",528, 480,
"png", NULL)
mSet<-PlotKEGGPath(mSet, "Pantothenate and CoA biosynthesis",NA, NA,
"png", 600)
mSet<-PlotKEGGPath(mSet, "Citrate cycle (TCA cycle)",528, 480, "png",
NULL)
mSet<-PlotKEGGPath(mSet, "Citrate cycle (TCA cycle)",NA, NA, "png",
600)
mSet<-PlotKEGGPath(mSet, "Glycine, serine and threonine metabolism",
528, 480, "png", NULL)
mSet<-PlotKEGGPath(mSet, "Caffeine metabolism",528, 480, "png", NULL)
mSet<-PlotKEGGPath(mSet, "Cysteine and methionine metabolism",528,
480, "png", NULL)
mSet<-SaveTransformedData(mSet)
```
